# A Neurocomputational Model of Stimulus-Specific Adaptation to Oddball and Markov Sequences

**DOI:** 10.1371/journal.pcbi.1002117

**Published:** 2011-08-18

**Authors:** Robert Mill, Martin Coath, Thomas Wennekers, Susan L. Denham

**Affiliations:** 1 School of Psychology/Centre for Robotics and Neural Systems, University of Plymouth, Plymouth, United Kingdom; 2 School of Computing and Mathematics/Centre for Robotics and Neural Systems, University of Plymouth, Plymouth, United Kingdom; Indiana University, United States of America

## Abstract

Stimulus-specific adaptation (SSA) occurs when the spike rate of a neuron decreases with repetitions of the same stimulus, but recovers when a different stimulus is presented. It has been suggested that SSA in single auditory neurons may provide information to change detection mechanisms evident at other scales (e.g., mismatch negativity in the event related potential), and participate in the control of attention and the formation of auditory streams. This article presents a spiking-neuron model that accounts for SSA in terms of the convergence of depressing synapses that convey feature-specific inputs. The model is anatomically plausible, comprising just a few homogeneously connected populations, and does not require organised feature maps. The model is calibrated to match the SSA measured in the cortex of the awake rat, as reported in one study. The effect of frequency separation, deviant probability, repetition rate and duration upon SSA are investigated. With the same parameter set, the model generates responses consistent with a wide range of published data obtained in other auditory regions using other stimulus configurations, such as block, sequential and random stimuli. A new stimulus paradigm is introduced, which generalises the oddball concept to Markov chains, allowing the experimenter to vary the tone probabilities and the rate of switching independently. The model predicts greater SSA for higher rates of switching. Finally, the issue of whether rarity or novelty elicits SSA is addressed by comparing the responses of the model to deviants in the context of a sequence of a single standard or many standards. The results support the view that synaptic adaptation alone can explain almost all aspects of SSA reported to date, including its purported novelty component, and that non-trivial networks of depressing synapses can intensify this novelty response.

## Author Summary

For processing real-life auditory scenes, it is not enough that auditory neurons code only for basic stimulus properties, such as frequency and intensity; at some point, these isolated properties must be woven into a pattern. Stimulus-specific adaptation (SSA), whereby neurons adapt to common stimuli but otherwise remain sensitive to other, rare stimuli, has been proposed as a low-level substrate for such abstract pattern processing. SSA has been previously investigated using ‘oddball sequences’ of tones, in which one frequency is common, the other rare. In this article, we present the first neurocomputational model of SSA and show that it can reproduce a wide range of published data. We also propose a natural generalisation of the oddball paradigm, based on Markov chains, which allows the experimenter to manipulate other characteristics of the sequence such the rate of switching. Finally, we show that a small network of neurons can distinguish novelty from mere rarity; e.g., a B stands out in the sequence ABAAA in a way that it does not in CBADE, even though it is equally probable in both. We demonstrate that cascades of depressing synapses can adequately encode this difference, whereas the simple adaptation-based models proposed to date cannot.

## Introduction

Natural acoustic environments play host to a wide variety of sounds that are either repetitive or follow a regular pattern. If an organism that inhabits one of these environments hears a repeating sound and does not react to the first few salient presentations, then it is unlikely that further repetitions will be behaviourally relevant. On the other hand, if the organism is to respond to changes in its environment, then it cannot adapt to stimuli indiscriminately; rather, it must remain sensitive to even small deviations from an established pattern. It is within such an evolutionary context that the brain has acquired stimulus-specific adaptation (SSA) mechanisms that operate across several time scales and sensory resolutions [1].

SSA in response to tone sequences has been measured in the spiking of single neurons at various stages of the auditory pathway, including the inferior colliculus (IC) in the rat [2], [3], medial geniculate body (MGB) of the thalamus in the mouse [4] and rat [5], thalamic reticular nucleus in the rat [6], and primary auditory cortex in the cat [7], [8] and rat [9]. It has been suggested [7], [8], [10] that SSA in single neurons lies on the path leading to the generation of *mismatch negativity* (MMN)–a frontocentrally negative-going deflection in the event-related potential [11], [12], evoked in response to violations of an established temporal sound pattern, including changes in frequency, intensity, duration and even the omission of an expected stimulus (for a recent review, see [13]). It is thought that MMN, in turn, may be implicated in the redirection of attention [14], maintain the representation of the auditory context [12], and contribute to auditory scene analysis [12], [15].

In this article we describe a neurocomputational model of SSA based on a small network of spiking neurons connected by dynamic synapses. The model components are all drawn from the literature [16]–[19] and are implemented without significant modification in order to keep free parameters to a minimum. In terms of its overall architecture, the model rests upon few anatomical assumptions, as it consists solely of a small number of homogeneous populations joined together in uniform patterns of connectivity, which could exist in the brain (e.g., all-to-all, sparse/random). The model requires feature-tuned inputs but does not require that these inputs be mapped topographically. As frequency selectivity in neurons is best understood, and most SSA studies to date have only manipulated frequency, the inputs of our model are tuned to frequencies. This study offers three distinct contributions to the ongoing discussion concerning stimulus-specific adaptation in single neurons: a new model of SSA that accounts for an array of experimental results; a description of a novel stimulus paradigm, accompanied by predictions from the model that can be tested experimentally; and an exploration of the effect of linking adapting processes in series on SSA and novelty detection in general.

It is sometimes remarked that the time scale of recovery from adaptation to tones measured in cortex is consistent with the time it takes cortical synapses to recover from synaptic depression [7], [8], [20]. Despite the strikingly suggestive similarity in the dynamics, and the availability of a light-weight model of a depressing cortical synapse [18], we are not aware of any modelling study to date that has explicitly attempted to bridge this explanatory gap: assembling these model synapses into networks with a view to replicating the results of SSA experiments. Here we undertake just such a study, taking as our primary data the results obtained by von der Behrens et al. [9] in the auditory cortex of the awake rat, which are presented in such a format as to be particularly conducive to the calibration of a model. A more general, theoretical treatment of the properties of networks constructed using this dynamic synapse model is given in [21]. Some mathematical results pertaining to SSA when viewed as an abstract computational process are discussed in [22].

Having configured the model to respond to oddball sequences in a manner consistent with the published physiological data, we then probe it with patterns of standards and deviants generated by first-order Markov chains [23], wherein the probability that a given tone is standard or deviant depends on its immediate predecessor. Oddball sequences actually constitute a specific subset of two-state Markov chains. Progressing to general Markov chains enables one to vary not only the probability of a deviant (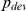), but also the probability of switching between deviants and standards (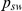); or, from another perspective, to control the degree to which deviants and standards “clump together” in the sequence, whilst maintaining their overall proportions. The model furnishes explicit predictions regarding the response of SSA neurons to tone sequences generated by Markov chains.

Finally, we examine serial arrangements of depressing synapses as a possible basis for certain types of novelty detection. This architecture is motivated by the fact that some neurons respond more vigorously to deviant tones if they are embedded in a background of a single standard frequency than if they appear as one of many, equiprobable random tones [7], [10]. At the very least, the difference in the responses is not so great as one would expect from a model based on adaptation within channels [24]. A similar sensitivity to novelty is also apparent in the mismatch negativity [25], [26]. The idea of a two-layer model rests on the plausible suggestion that the pre-synaptic inputs to some depressing synapses themselves undergo adaptation due to synaptic depression elsewhere.

In the current study, we found that cross-channel adaptation within a single layer of depressing synapses was sufficient to account for the excess response to deviants embedded in a single standard provided that 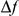 was large enough. However, introducing two layers of synaptic depression in series enhanced the effect, in that this excess response was larger, and the 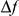 required to elicit the effect was smaller. In summary, on the one hand, our results support the case for an explanation of SSA based solely on adaptation, at least as far as frequency is concerned. On the other hand, commentators that adopt an adaptation-based interpretation of SSA tend to speak exclusively in terms of the depression or fatigue associated with afferents, whereas we demonstrate that linking depressing synapses in series (and, in principle, recurrently) can dramatically modify these effects.

## Methods

**Figure 1:**
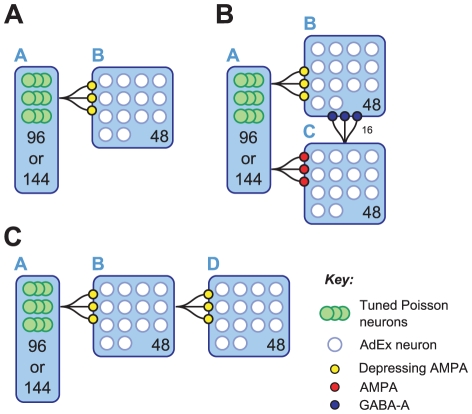
SSA model architectures. The blue boxes depict populations, and the figures printed inside state the number of units (or Poisson groups). The number of sub-populations in population A depends on whether the task is two-tone (96) or multi-tone (144). A) AB model consisting of a single layer of depressing synapses. B) ABC model introduces an inhibitory population. C) ABD population consisting of two layers of depressing synapses. The synaptic pathways drawn between populations stand for all-to-all connectivity. An exception is 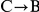: each unit in B receives 16 synapses at random from units in C.

In this section, we first describe the individual components that constitute the model, and then explain how these components are assembled to form networks containing units that exhibit SSA. We then discuss the time-varying patterns supplied as input to these networks, which are taken to represent the kinds of tone stimuli used in physiological SSA experiments. The neurocomputational models presented in this article are constructed from spiking units partitioned into populations, labelled A to D. We consider three types of network, and designate each according to the populations it contains: the AB model, the ABC model and the ABD model. These networks are illustrated schematically in Figure 1 and are described in greater detail below. Some results from an ABCD model, which contains all four populations, are included in Supplementary Text S1.

### Model Components

#### Spiking neuron models

The units in population A are independent Poisson processes, whose firing rates are modulated by the input stimulus. The units in populations B to D implement the adaptive exponential integrate-and-fire (AdEx) model proposed in [16], which incorporates sub-threshold and spike-triggered adaptation currents. Every AdEx model uses the parameters listed in (Table 1 in [16]).

#### Dynamic synapses

The synapses in the model fall into three classes: fast excitatory, fast inhibitory, and fast excitatory with rapid depression and slow recovery. Fast excitatory and inhibitory synapses are based, respectively, on the simplified kinetic models of the AMPA/kainate and 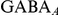 receptors described in [17], and we adopt the parameter sets provided there.

The depressing synapse model combines features of the AMPA synapse model from [17] and the model presented in [18]. It assumes that a unit supply of resources is divided amongst three states: *recovered* (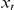), *effective* (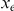) and *inactive* (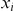). Initially, 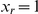 and 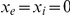. The system of equations governing the flow of transmitter between states is similar to that found in [18]:
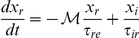


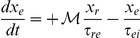


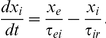
Following a pre-synaptic action potential, 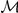 is set (or reset) to one for a duration of 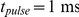; afterwards, it returns to zero. In these equations, 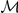 refers to a quantity analogous to that defined in [18] as 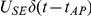. Whereas the model in [18] uses a delta function to represent the effect of a pre-synaptic spike, this model and [17] use a brief, square pulse (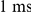). The time constants 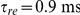 and 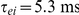 are taken from [17], and control the rate at which recovered transmitter substance becomes effective, and effective transmitter substance becomes inactive, respectively. The third time constant, 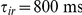, controls the rate at which inactive substance is recovered and is taken from Figure 1B in [18]. Note that by setting 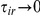 one obtains the non-depressing version of the AMPA synapse. The excitatory post-synaptic current for the depressing synapse is then proportional to the fraction of substance that is effective:
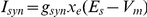
where 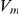 is the post-synaptic membrane potential, 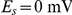 is a reversal potential [17], and 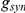 is an overall synaptic efficacy.

#### Noise sources

Altogether, three sources of noise may be identified in the model. First, upon initialisation, the parameters of every synapse in the model (time constants, 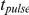, synaptic efficacies, reversal potentials) are perturbed by multiplication with log-normal random variables [27] (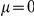; 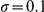). The neuron parameters are not perturbed.

Secondly, every AdEx neuron is subject to an *in vivo*-like fluctuating noise current to simulate synaptic background activity. The noise model and its parameters are taken from eqn. 2; Table 1, col. 1 in [28], with two exceptions: the overall magnitude of the current is scaled to compensate for the change in surface area between the neuron modelled in [28] and that modelled here [16], [19]. The standard deviation of the excitatory conductance, designated 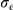 in [19], is set to one of two values, depending on the experiment. For the AB and ABC models, 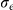 is hand-tuned to 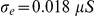 to yield a mean firing rate of approximately 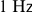, typical of high spontaneous activity in auditory cortex. For the ABD model, 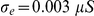. This is the original value used in [17] and causes membrane potential fluctuations, but few spontaneous spikes. The third source of noise is due to variability in the spiking of the Poisson neurons between repeated trials.

The level of spontaneous activity varies amongst SSA studies [5], [9]. A high level of background noise was incorporated into the models to ensure that the SI values obtained from the model were conservative (i.e., likely, if anything, to be higher in a cleaner model), and also to militate against the possibility of obtaining results that required delicately chosen synaptic weights.

### Input Population (A)

Population A comprises sub-populations of Poisson neurons, each of which fires at a rate that depends on the frequency of the input tone. The best frequencies of the sub-populations are spaced uniformly on an octave scale. The number of sub-populations and the range of octaves spanned is task-dependent: two-tone tasks utilise 96 inputs spanning a range of 2 octaves; multi-tone tasks utilise 144 inputs spanning a range of 3 octaves. The firing rate (Hz) of sub-population 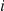 with best frequency 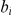 in response to tone frequency 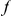 has the form of a raised Gaussian profile,
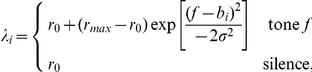
where 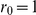 is the spontaneous firing rate in the absence of a signal; 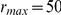 is the maximum firing rate, elicited when the tone and best frequencies coincide; and 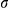 controls the width of the tuning curve.

**Figure 2:**
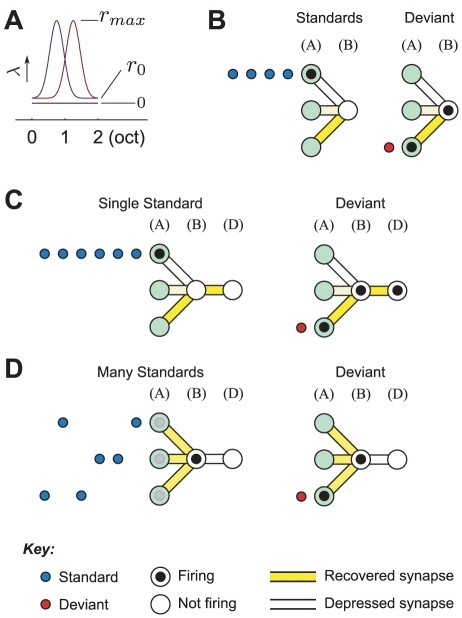
Operation of the AB and ABD models. A) Tuning profiles of two Poisson neurons spaced 0.5 octaves apart. B) Graphical explanation of SSA in the AB model. The 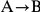 synapses associated with the standard frequency are depressed, so that a neuron in B responds less to standards (blue), but remains sensitive to deviants (red). C, D) Graphical explanation of SSA in the ABD model in response to a single standard followed by a deviant (C), and many standards followed by a deviant (D).

As a measure of bandwidth, we take the separation, in octaves, between the frequencies that evoke firing rates half-way between the maximum and spontaneous rates, and denote this quantity 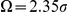. Unlike stimulus parameters, which can be chosen to match the original SSA experiments exactly, the tuning of the putative input channels can, at best, only be inferred from the results of the SSA experiments themselves, or estimated in line with other experimental data. We typically set 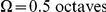 in this study, which we consider to be conservative, given the tuning width of certain neurons in the inferior colliculus [29] and fibres at the auditory periphery [30]. Alternative values for 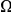 are also investigated, however. Figure 2A depicts the overlap between two tuning curves with best frequencies separated by half an octave.

### AB Model

The AB model is the simplest instance of an adaptation-based model that exhibits SSA. It consists of two populations labelled A and B (see Figure 1A). The computations relevant to SSA are effectively performed by a single, feed-forward layer of depressing synapses (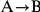).

Population B consists of 48 AdEx neurons, each of which receives a connection from a distinct Poisson neuron in every sub-population of A via a depressing, excitatory synapse. Thus population A contains 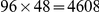 or 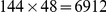 Poisson neurons, depending on whether the experiment is two-tone or multi-tone, respectively. It is the depression of the 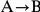 synapses which guarantees the basic behaviour required of the model, namely, that the responses in B reduce if the same tone is presented repeatedly, but recover if another tone is presented. This scenario is presented diagrammatically in Figure 2B. The degree of overlap in the tuning of the Poisson inputs determines how SSA varies with the frequency separation between the tones. When 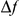 is small, the synaptic resources associated with the standard and deviant frequencies coincide to a greater extent, and the SSA measured is smaller.

### ABC Model

The ABC model extends the AB model by adding an inhibitory population, C, consisting of 48 AdEx neurons, and two additional synaptic pathways, 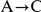 and 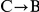 (Figure 1B). The connectivity of the 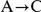 pathway is identical to that of 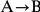, described above, with the exception that the synapses involved do not depress. Each unit in population B receives input from sixteen randomly-chosen units in population C via fast, inhibitory synapses, which collectively form the pathway 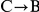. As in the AB model, SSA is sought in population B.

In this model, the indirect pathway 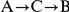 does not participate in the generation of SSA. Rather, the tonic inhibition of population B ensures that spontaneous activity is minimised, so that spiking activity reflects the input signal, not the background noise. Peri-stimulus time histograms (PSTH) from a study of SSA in the awake rat [9], show a transient response at the tone onset, followed by a period of spiking below the spontaneous rate, suggestive of inhibition, which lasts for the duration of the tone (see Figures 1A and 3A in [9]; see also Results).

**Figure 3:**
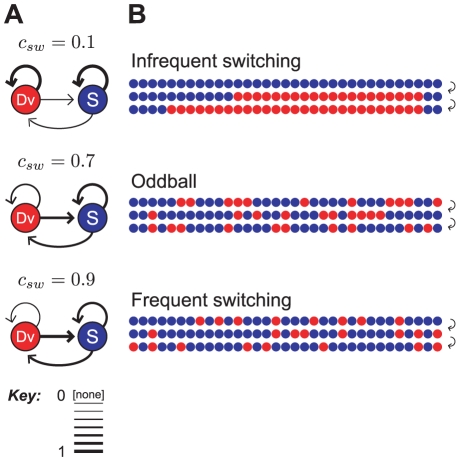
Two-state Markov chains. State transition diagrams (A) and example sequences (B) for three two-state Markov chains with different scaled switching metrics. The transition probabilities are represented using line thickness (see Key). Standards and deviants are indicated in blue and red, respectively. Each block shows ninety-nine tones wrapped onto three lines.

In summary, the SSA responses in the ABC model are essentially generated in the same way as those in the AB model, namely, through the depression and recovery of the 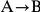 synapses. There is, however, a difference in the resultant firing patterns. In the AB model, activity in population B persists throughout the tone, until the 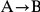 synapses are depressed to the extent that the units can no longer reach threshold. In the ABC model, in contrast, the neurons receive a strong, delayed, shunting inhibitory input, which suppresses both spontaneous and stimulus-driven spiking. Thus, if a neuron in population B is to fire at all, the excitatory component from population A must cause it to reach threshold in the short time window before it is inhibited. An appropriate balance of excitation and delayed inhibition leads to binary spiking, i.e., the tendency to respond to a stimulus with either no spikes or one spike, which is observed in auditory cortical neurons in general [31], and also in SSA studies in cortex [9] and MGB [5]. Synaptic depression weakens the excitatory contribution to the post-synaptic potential and effectively turns this binary response from ‘on’ to ‘off’.

### ABD Model

The ABD model extends the AB model by adding population D, which consists of 48 AdEx neurons, and an excitatory synaptic pathway, 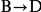. There are no inhibitory populations in this model. The units in population D receive input from population B only, via depressing synapses, connected in an all-to-all pattern (Figure 1C). Our primary interest is SSA in population D, although SSA is also present in population B.

Whilst several authors have suggested adaptation on the inputs to a neuron as the mechanism whereby SSA is generated [7]–[9], none have considered the properties of a network consisting of a cascade of depressing synapses. The ABD model is used to investigate the simplest instance of such a network, in which there are just two depressing pathways (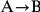; 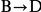). The 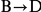 pathway has a recovery time constant of 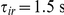 [7], [32]. The synaptic weights are 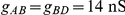.

The original motivation for the ABD model was the suggestion that the responses obtained for deviants embedded in a single standard exceeded those obtained for the same deviants embedded in a “many standards” control condition [10]. We elaborate on the descriptions of these protocols below. Here it will suffice to sketch the intuitive difference in the stimuli and the behaviour required of the model. If deviant tones are presented against a background of a single, repeating standard frequency, then they are *conspicuous*, and the model should respond. However, if the same deviant frequency appears as one of many equiprobable random tones, then it is no longer conspicuous, it is simply one tone amongst many, and the model should not respond. In summary, the model must respond to the *novelty* of the tone, not simply its *rarity*–which is the same in both conditions.

Figure 2C–D illustrates how the two-layer architecture can make this distinction. Figure 2C shows how the model responds to a deviant embedded in a single standard. A repetitive standard (left) causes the synapses associated with that frequency to depress, and the neurons in population B stop firing. Because the activity in population B is low, the 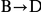 synapses do not depress. When a deviant tone is presented (right), there is a recovered synaptic pathway leading from population A to D, via B, and the neurons in population D respond.

Now we consider the many standards configuration. Figure 2D (left) depicts the presentation of many standards. Because the frequencies of the standards vary, there is usually time for the 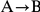 synapses to recover between presentations. As a consequence, the average response in population B is high, and 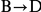 synapses are depressed. Now, when the nominal deviant tone is presented (right), there is no longer a complete pathway of recovered synapses leading from A to D, and the neurons in population D are silent. The units in population D of the ABD model react to deviants in an appropriate context-dependent manner, whereas the units in population B do not. In closing, we emphasise that the binary distinctions firing/not firing and depressed/recovered are drawn for the benefit of the illustration. In the model, we seek only differential effects consistent with this general behaviour.

### Stimulus Configurations

#### Oddball sequences

Oddball stimuli are sequences of tones consisting of two frequencies, 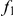 and 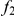 (Hz), one of which is deviant, and the other standard. The ratio of standards and deviants is controlled. The frequencies are presented to the model equally-spaced on an octave scale around the centre of the input range. Each oddball sequence is presented twice: 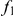 and 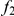 are swapped in the second presentation, but the pattern of standards and deviants is preserved. It is necessary to present the same frequency in a standard and deviant context in order to control for any frequency preference associated with the neuron.

As in [7] and elsewhere, 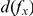 and 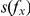 refer to the mean spike count elicited in response to frequency 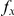 when presented as the standard or deviant, respectively. The degree of stimulus-specific adaptation is quantified using various *SSA indices* (SI) [7]. The *frequency-specific* SI is a normalised measure of the difference in responses to 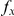 when deviant and standard:
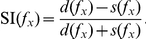
The SI is confined to the interval 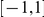. A larger SI corresponds to greater excess in the deviant response over the standard, and in fact, when the two responses are very close, 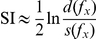. SSA is absent when the SI is not significantly positive. The *neuron-specific SI* quantifies the overall level of SSA that a neuron exhibits, and it has a similar definition:
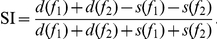
The term “SI”, without qualification, denotes the neuron-specific SI. (For discussion of an alternative version of the oddball paradigm, called the “switching oddball design”, see [8] and Supplementary Text S1.)

#### Two-state markov chains

Oddball sequences have been widely used to investigate SSA; but little consideration has been given to the possibility of employing *Markov chains* [23] in a similar capacity–Markov chains being a broader class of random process, to which oddball sequences belong as a special case. If one designates states 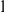 and 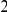 of a two-state Markov chain as ‘deviants’ and ‘standards’, respectively, then the transition matrix for an oddball sequence can be written
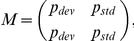
where each element, 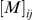, relates the probability of transiting from state 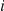 to state 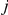. The stationary distribution for this Markov chain is the vector 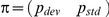, i.e., 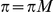. The probability of switching between states depends on the probability of a deviant; specifically, 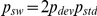.

Two-state Markov chains offer a way to decouple the probability of switching from the probability of a deviant. This generalised Markov chain has two degrees of freedom, and its transition matrix has the form
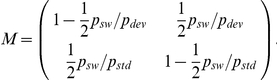
As the maximum valid choice for 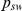 depends on 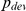, it is convenient to define a *scaled switching metric*, 
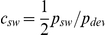
, which, for a given 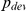, expresses how often a Markov chain transits from one state to the other, as a value between zero (never switches) and one (switches at highest possible rate). Figure 3 shows state transition diagrams and example realisations of Markov chains where 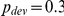 is held fixed and 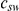 is varied. The response of the ABCD model to three-state Markov chains is discussed in Supplementary Text S1.

#### Block, random and sequential stimuli

Multiple tone frequencies are routinely used to evaluate the frequency-response areas of neurons and have also been used to assess stimulus-specific adaptation [2], [7]. Pérez-González et al. [2] measured the responsiveness of neurons in the rat IC to one hundred-tone sequences, consisting of ten frequencies repeated ten times. In this protocol, the tones are presented in three configurations: *block*, *sequential* and *random*. In block mode, tones of identical frequency are presented in blocks, ascending from the lowest frequency to the highest. In sequential mode, an ascending, stepwise series of tones is repeated ten times. In random mode, the tones are ordered randomly.

#### Deviants amongst many standards

Oddball experiments cannot, by themselves, adjudicate the question of whether the enhanced response to a deviant, if present, is due to its *novelty*–the fact that it stands out against a uniform background–or simply its *rarity*. Previously, to address this issue, the “deviant amongst many standards” protocol has been used as a control condition for MMN oddball experiments [25], [26]. A sequence of many, equiprobable tones is presented, which is constructed in such a way that the deviant frequency still appears in the same positions as it did in an oddball sequence. The average responses to deviants presented in the two contexts are then compared to delineate the effect of the context on the processing of the same sound. (Note that in the many standards condition, the term ‘deviant’ is employed in a nominal sense, as it refers to the true deviant in the corresponding oddball sequence.) The signal is enhanced when the deviant tone is presented against a background of a single standard (the difference is termed the “true MMN”), and the same is true for the spiking responses of single cortical neurons [7] (see discussion in [10]), which we aim to model here.

## Results

### AB Model

This section reports the response of the AB model to oddball sequences only. The responses of the AB model to other types of sequence are discussed in the ABD Model section.

#### Oddball sequences

**Figure 4:**
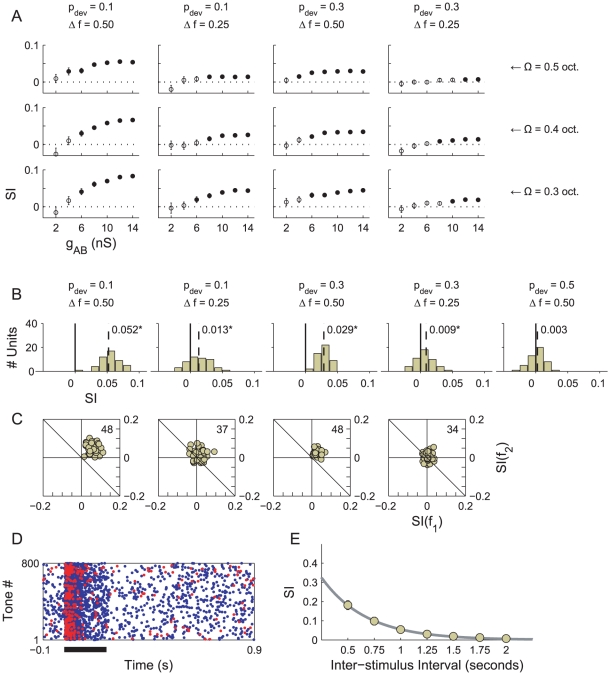
SI values obtained from the AB model. A) SI values measured in each condition (column) as a function of 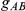 (abscissa) and 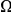 (row). Filled circles indicate an SI that differs to zero significantly (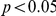, signed rank test). B) Histograms of the neuron-specific SIs collected over the 48 neurons in population B for five stimulus conditions, which are listed above. The median SI is printed in black on each set of axes and marked using a vertical dotted line (*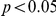, signed rank test). C) Scatter plots of the frequency-specific adaptation indices for 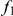 and 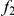 obtained in the model. The number of data points out of 48 that fall strictly above the marked diagonal is printed in the top-right corner of each plot, respectively. SSA is deemed present when this figure represents a significant majority of the neurons. D) Raster plot showing the response of an exemplary neuron in population B over 800 trials. Red and blue dots plot spikes in response to standards and deviants, respectively. Thick line indicates tone duration. E) SI as a function of onset-to-onset ISI. The thick, grey line provides an exponential fit.

In the first set of experiments we tested the AB model using eight-hundred tone oddball sequences. The SI was measured for four conditions, setting 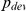 to either 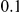 or 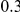, and setting 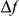 to either 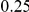 or 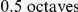. In all conditions, the tone rate was 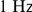, and the tone duration was 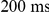. Two parameters of the model were also varied: the 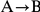 synaptic weight (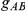), and the bandwidth of the tuning curves in population A (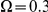, 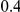 or 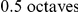). The results are plotted in Figure 4A.

We first consider the effect of the 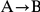 synaptic conductance on the SI. In all conditions, as 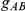 is increased, the SI increases and forms a plateau. When 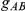 is set to zero, there is no synaptic connectivity between the populations. In this case, the SI values must be derived entirely from spikes due to spontaneous activity, and the expected SI value is zero. As 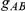 increases, activity in population B is driven by the stimulus to a greater extent. The SI value consequently starts to increase and eventually differs from zero significantly (solid markers: two-sided Wilcoxon signed rank test, 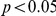). Once the signal-related activity is strong enough to overpower the noise background, a larger synaptic weight increases the overall spike counts (not shown) but does not affect the SI appreciably. A synaptic weight of 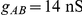 produces high firing rates in the 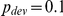/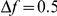/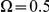 condition. The peak instantaneous firing rate at the onset of a deviant is 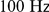, which reflects a burst of spikes in quick succession; the tonic firing rate is 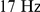 (see Figure 4D). In order to retain realistic firing rates, we did not test stronger synaptic weights.

We next examine the influence of the input bandwidth (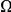) on the SI. Each selection of bandwidth corresponds to a row in Figure 4A. Using a narrower bandwidth increases the SI in each condition over the range of 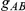 used. Due to spontaneous noise in the system, the SI does not increase at every data point; however, the effect of bandwidth on sets of points taken as a whole is readily apparent. This relationship between bandwidth and SSA is to be anticipated, given the improvement in resolution that follows from a decrease in 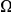. However, one additional complication should be noted before proceeding. In the AB model, a narrower bandwidth also leads to a lower level of excitation in population B because fewer units in population A are activated. This implies that the improvements in SI which result from constricting the bandwidth, evident in Figure 4A, are smaller than they would be if 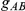 were concurrently increased to compensate for the drop in net excitation.

We centre our discussion of the effect of 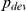 and 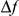 on the SI around a particular results set, presented in Figures 4B and 4C, in which the model parameters were held fixed (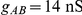 and 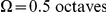). Figure 4B provides histograms showing the SIs measured in population B for five stimulus conditions: the four conditions listed above, plus an additional control condition, in which the two tones are equiprobable, i.e., 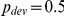. The influence of basic oddball sequence parameters upon SSA is consistent with physiological studies [3], [7], [9]: increasing 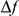 increases SSA, as does decreasing the probability of a deviant. SSA is insignificant in the control condition, according to a two-sided Wilcoxon signed rank test (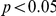).

Figure 4C shows scatter plots of the frequency-specific SIs for 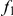 and 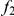. Plots of a similar kind feature in [3], [5], [7], [9] and provide an alternative perspective on the data used to produce the histograms of neuron-specific SIs, and specifically, whether those SIs derive from adaptation to 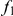 or 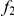. The results here are typical of those presented in all the cited studies, including [9]. Data points are asymmetrically distributed around the 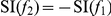 diagonal (drawn) when SSA is exhibited and extend further into the positive quadrant when SSA is stronger. The distribution around the perpendicular diagonal, 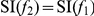 (not drawn), is symmetric, indicating that SSA is not due to adaptation to one frequency more than the other [3], [5]. This symmetry is unsurprising if the depression characteristics of synapses are independent of the tonotopic location of their pre-synaptic efferents, as they are in this model.

#### Effect of noise and tone rate

The SI values measured in population B, though significant, are much lower than those typically observed in physiology [3], [7], [9], although using a smaller value for 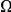 leads to some improvement (Figure 4A). The raster plot in Figure 4D shows the response of a neuron in population B over 800 trials of the 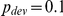 and 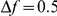 condition. The spontaneous noise background produces many spikes that are independent of the signal, and this in turn leads to lower SI values. Simply disabling the noise and repeating the experiments was not a practical course of action. The spontaneous spikes are brought about by membrane potential fluctuations (see Methods), the presence of which influence the effectiveness of synaptic currents [28]. Consequently, disabling the noise would mean a wholesale re-adjustment of synaptic weights. In other words, there was no straight-forward, controlled way to compare the performance of the model with the noise switched on and switch off. Another factor which could result in small SI values was the slow tone rate used (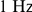). Figure 4 shows the SI values obtained for different inter-stimulus intervals (ISI; the reciprocal of the tone rate) when 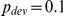 and 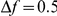 were fixed. Larger SI values were measured for oddball sequences with short ISIs. The issues of noise and tone rate are discussed further in the next section.

### ABC Model

#### Oddball sequences

The next task was to calibrate the parameters of the ABC model so that the neurons in population B exhibited similar adaptation characteristics in response to oddball sequences to units in the auditory cortex of the awake rat, according to the results reported by von der Behrens et al. [9]. The ABC model was tested with oddball sequences using 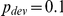, 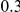 or 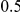 (control), and 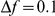 or 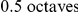. The tone rate and tone duration were initially held fixed at 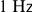 and 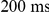, respectively. A total of 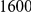 tones were presented in each oddball condition: 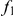 was deviant in the first block of 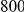; 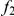 was deviant in the second block of 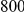. These stimulus parameters are identical to those used in the evaluation of the AB model described earlier and to those specified in [9].

Von der Behrens et al. [9] provide figures offering several perspectives on the same data. These include histograms and scatter plots of SIs, peri-stimulus time histograms and spike raster plots that display activity on a fine time scale, and graphs showing the response to deviant tones conditioned on various recent tone histories. The principal aim was to obtain a qualitative match to their data, as presented in these various formats, and wherever possible, to achieve a quantitative fit as well. This section reports the outcome of this process and includes some additional results.

Some useful numerical data for our purposes consisted in the median SI values added to the histograms in Figure 4 of [9]. It was observed that these SI values exhibited the same partial ordering in 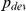 and 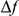 as those in Figure. 4B and were on the same order of magnitude (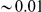, max. 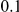). Thus rather than engage in a fine-grained, high-dimensional parameter search, which would consume several weeks, we hand-tuned the parameters, starting with the loose fit already obtained in the AB model and proceeding from there using heuristics. We initially set 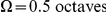 and 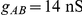, leaving two parameters free: 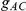 and 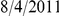
–the synaptic weights on the 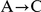 and 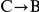 pathways. These two parameters control the same notional quantity, namely, the inhibitory effect of population A on population B. The first increases the firing rate in population C; the second increases the inhibitory efficacy of each spike. It was therefore appropriate to fix one arbitrarily and vary the other. Accordingly, 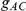 was set to 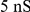, which was sufficient to generate moderate, tonic spiking activity in population C (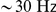). 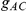 was then progressively adjusted until 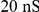 was reached, at which point the median SI values were close to those in [9], and the PSTH exhibited similar qualities to those obtained by physiologists (see next section).

**Figure 5:**
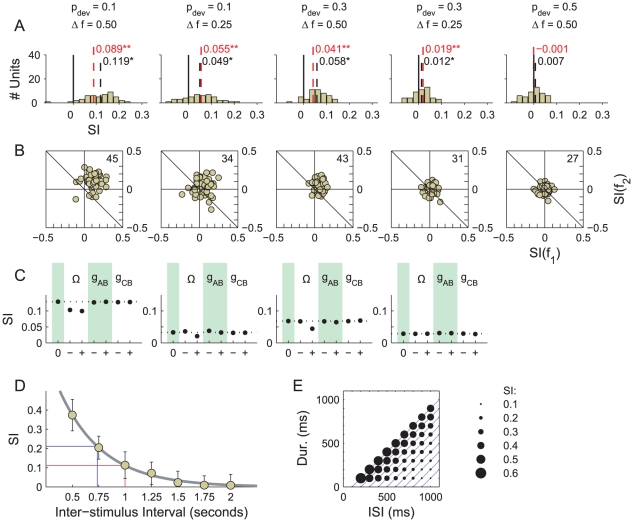
SI values obtained from the ABC model. A) Histograms of the neuron-specific SIs collected over the 48 neurons in population B for five stimulus conditions, which are listed above. The median SI is printed in black on each set of axes and marked using a vertical dotted line (*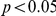, signed rank test). The corresponding median SI from (Figure 2 in [9] is superimposed in red (**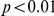). B) Scatter plots of the frequency-specific adaptation indices for 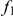 and 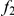 obtained in the model. The number of data points out of 48 that fall strictly above the marked diagonal is printed in the top-right corner of each plot, respectively. C) Effect of small parameter changes (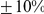) on the SI. The condition is printed at the top of each column. D) Mean SIs obtained from oddball experiments, in which the ISI was independently varied, and 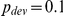 and 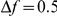 were controlled. Error bars denote standard deviation. The thick, grey curve shows an exponential fit to the data. The two read-offs correspond to the ISIs used by von der Behrens et al. (1 second; red) and Ulanovsky et al. (736 ms; blue). E) Bubble plot showing the mean SIs obtained from oddball experiments, in which the ISI and tone duration were varied. Note that unit-slope diagonals (light blue) are iso-contours for time to recovery (offset-to-onset).

Figure 5A shows the set of SI histograms obtained for the final choice of parameters in response to the five oddball stimulus conditions. The plots compare the median SIs obtained from the model (black dotted lines) with those published by von der Behrens et al. (red dotted lines). A good visual fit to the published SI distributions (Figure 4A in [9]) is obtained, both in terms of central tendency and spread. One notable difference between the physiological and modelling results relates to the ordering of SIs in those conditions where the parameters are opposed in their effects, i.e., deviants are rare but close in frequency to standards, or deviants are well-separated in frequency from standards but are common. In [9], the step that makes deviants more common (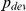 from 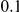 to 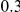) reduces the SI to a greater extent than moving them closer in frequency to standards (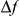 from 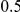 to 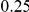), and the same pattern is observed in SSA measurements made in IC [3]. However, the reverse is true for the model, where reducing 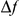 has a greater impact on SSA (Figure 5A, cols. 2, 3). This particular discrepancy could be addressed by choosing a smaller bandwidth parameter, 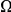, to improve the SI values in both of the 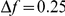 conditions, which are slightly too low in the model. The effect of small bandwidth changes on the model are examined shortly.

Figure 5B presents scatter plots of frequency-specific SI values, which should be compared with Figure 4B in [9]. The plots are visually similar in terms of the extent to which the points scatter in the (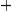,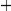) direction, which is to be expected, given the similarity in the SI histograms noted above. The plots differ, however, in that the spread of points along the reverse diagonal appears slightly greater in von der Behrens et al.'s data. This suggests the presence of some neurons for which adaptation is strong for one frequency and weak for the other. Although we randomly perturbed synaptic weights and other parameters to ensure individual characteristics for each neuron (see Methods), it nevertheless appears that adaptation to 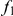 and 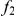 remain roughly equal in our model, and that the spread of SI values is chiefly due to noise.

Finally, we analysed the robustness of the model with respect to the parameters in order to ensure that the SIs obtained did not rely on a fine-balanced set of synaptic weights. We perturbed each of the three main parameters of the model by 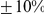 and investigated the effect upon the (mean) SI measured in each condition. These parameters were the bandwidth 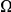, the excitatory weight 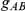, and the inhibitory weight 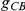. The outcome of this procedure is shown in Figure 5C. In each subplot, the left-most of the data points and the dotted horizontal line indicate the unperturbed SI. It is evident that changes in the synaptic weights do not affect the SI considerably. The SI is most affected by changes to the input bandwidth, 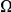. This can perhaps be explained by the fact that the bandwidth affects both the resolution of tones and the total excitatory input to population B. Using a narrower bandwidth (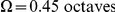) raises the SI value for the 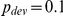 and 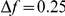 condition slightly.

#### Tone rate and duration

In order to investigate the effect of tone rate upon SSA, we presented oddball sequences with 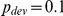 and 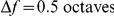 to the ABC model with different inter-stimulus intervals. The tone duration was fixed at 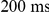. Figure 5D plots the SI measured as a function the ISI used. There is a clear trend showing that a shorter interval between tones–that is, a faster tone rate–results in greater SSA. This can be understood in terms of the rate at which the 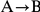 synapses recover from depression: a shorter ISI provides less time for the synapses to recover, which reduces the mean response to standards and increases the SI. At the opposite extreme, during very long ISIs, the synapses undergo a complete recovery between tones, and SSA disappears.

The influence of the tone rate upon SSA has already been explored experimentally in several auditory areas (IC [2], [3]; MGB [4], [5]; cortex [7]). The results, where clear, tend to reveal a positive correlation between tone rate and SI. Von der Behrens et al. [9] suggested that the SI values they recorded in cortex were smaller than those obtained by Ulanovsky et al. [7], [8] because they used a slower presentation rate (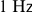; 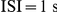), and their measurements were from an awake, rather than an anaesthetised, animal. In order to explore this proposal, we fitted an exponential curve to the data points in Figure 5D and read off the SI value expected in response to the tone rate used by Ulanovsky et al. (1.36 Hz; 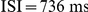). The result was an SI just above 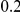
–very close to the mode of the SI histogram published Figure 2C, col. 2 in [7] for a comparable oddball sequence. This suggests that the differences in the cortical responses measured by von der Behrens et al. and Ulanovsky et al. are principally due to presentation rate.

We next examined the effect of tone duration on SSA. We expected that most synapses would depress within a few milliseconds of the tone onset (see switching oddball discussion above), and that any effect of tone rate on SSA would thus be indirect; that is, shortening the tone would decrease the SI only by virtue of lengthening the silent interval between the offset of one tone and onset of the next. The SIs obtained for various ISIs and tone durations, shown in Figure 5E, support this conclusion. Each unit-slope diagonal in this coordinate space corresponds to a given time to recovery, and it can be seen that the bubbles along these diagonals are approximately equal in size. The smaller SIs are along those diagonals which correspond to longer times to recovery. A residual increase in SI is also apparent for shorter tone conditions represented along the top diagonal. A key question for calibrating future models concerns how tone duration contributes to SSA. If increasing the tone duration and ISI by the same amount does not affect SI, then it is likely that synapses depress rapidly and remain depressed throughout the tone duration–which is the case in this model. If increasing the tone duration and holding the ISI fixed does not affect SI, then one may conclude that depressing synapses contribute only to the phasic response of the neuron.

#### Peri-stimulus time histograms

Another concern was the response of the model on shorter time scales, such as that revealed by PSTHs averaged over the course of individual tones. Some qualitative features of the responses in Figure 3 in [9] sought included: the presence of spontaneous spiking noise, a phasic response at the tone onset, a drop below spontaneous activity throughout the duration of the tone, and a recovery to the spontaneous rate of firing at the offset. Little effort was undertaken to match the spike counts from [9] absolutely, as those data mixed spike counts from single-unit and multi-unit recordings.

**Figure 6:**
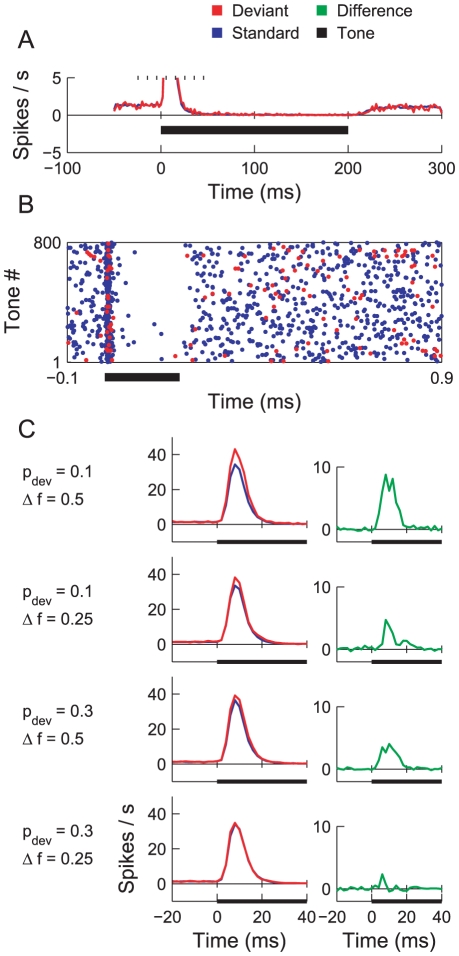
PSTHs from the ABC model in response to tones in an oddball sequence. A) Average, tonic firing rate of a neuron in population B during silence and tone input (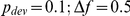), showing the effect of inhibition on the spontaneous rate during a tone (thick line). The phasic portion has been clipped. B) Raster plot of the spikes from a single neuron in population B during standard and deviant trials. C) PSTHs (left column) showing the onset responses for standard (blue) and deviant (red) tones in different oddball conditions, and the excess in the response to a deviant over that to a standard (right column, green). The analysis bin width in all PSTHs is 2 ms.

Figure 6A plots the PSTH averaged over 1600 tones and 48 units in the 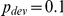 and 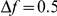 condition, concentrating on the changes in the sustained firing rate. The mean spike rate in the absence of a signal is about 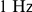; a burst accompanies the onset of the tone, and this is followed by a period of inhibition. The time courses of the phasic response and the recovery from inhibition (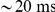) are similar to those published in Figure 3A in [9]. It should be noted that although Figure 6 gives the impression of a 20 ms burst of spikes, model neurons in fact typically fire once, if at all, and the breadth of the PSTH peak derives from variation in the timing of single spikes. Figure 6B is a raster plot showing the spikes emitted by a single neuron in population B.

Figure 6C (left column) overlays the PSTHs computed for the onset portion of standard and deviant tones in the four non-control oddball conditions. Changes in the oddball parameters, and between standard and deviant, appear to impact only the height of the PSTH peak; its shape and duration are unaffected. Waveforms plotting the difference between the deviant and standard PSTHs are shown in right-hand column of Figure 6C (compare Figure 3B in [9]). The largest and smallest difference waveforms correspond to the highest and lowest SI conditions.

The suppression of noise in population B due to inhibition leads to larger SI values in the ABC model than in the AB model, even though the period available for integrating signal-driven spikes is much shorter in the former, as a comparison of Figures 4D and 5B reveals. This points to the possibility of a noise-reduction role for inhibition, which comes into effect during periods of stimulation.

#### Recent tone history

**Figure 7:**
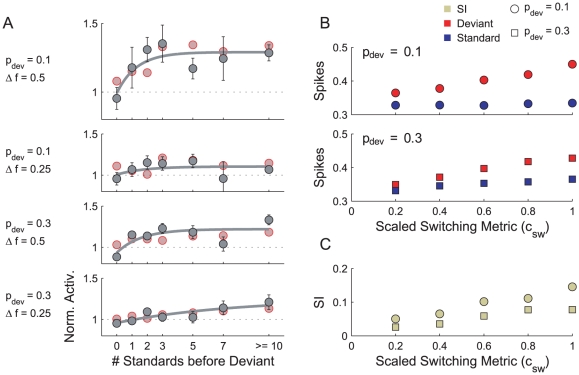
Adaptation rates in the ABC model and response to two-state Markov chains. A) Each marker plots the normalised mean response to a deviant as a function of the number of standards since the previous deviant. The normalisation is with respect to the mean response to all tones in that condition (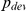, 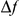). Error bars plot the standard error of the mean. Deviants preceded by ten or more standards are collected into a single data point. The solid curves are exponential trends fitted by a non-linear, least-squares regression. The results of von der Behrens et al. are plotted in red (measured from Figure 4 in [9]). B) Spike counts elicited by standards and deviants as a function of 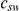, shown for 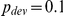 (upper) and 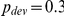 (lower). C) SIs measured as a function of 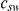.

The SI provides a measure of stimulus-specific adaptation over an entire oddball sequence, but it does not convey any information concerning how the response to a deviant tone is affected by the immediate history of tones (unless one initially assumes a model for how SSA comes about). To address this issue, following von der Behrens et al., Figure 7A plots the mean spiking activity in response to a deviant, conditioned on a fixed number of immediately preceding standards, and normalised by the mean response to all tones in the sequence. The results are to be compared with those in Figure 4 in [9] and have been set out in a similar format.

A good match to the physiological data has been achieved in at least three regards. First, in both figures, the deviant response is shown to increase with the number of preceding standards, and this trend is arguably an increasing form of exponential decay (see fitted curves). Secondly, the data are on the same order of magnitude, and assuming an exponential trend, the asymptotic responses (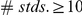) fall into similar ranges: the largest asymptote (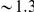) is seen in the high SI condition (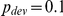 and 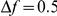), and smaller asymptotes (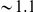 to 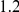) are reached in the other three conditions. The third remark relates to the rise times of the trends. In the high SI condition, the deviant responses do not change after about three standard tones. In the low SI condition (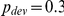 and 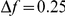), the rise time is slowest, to the extent that, on both figures, the exponential curve appears almost linear over the range of interest.

#### Markov sequences

The model and the experimental evidence [9] make it clear that the response to a tone is affected by the tones which immediately precede it, and it is possible that the SI value represents the accumulation of these local history effects. The probability of a deviant being preceded by another deviant in an oddball sequence is always 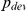. Generalising the oddball sequence to a two-state Markov chain provides a means of manipulating the probability of standards and deviants following one another, whilst maintaining the overall proportion of each (see Methods).

We next used the ABC model to predict how the neurons in the von der Behrens et al. study would respond to patterns of standards and deviants generated by a two-state Markov chain. The tone duration and tone rate were held fixed at 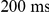 and 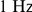, respectively. Each sequence comprised 1000 tones and was presented twice, with deviants and standards exchanged in the second presentation. In order to best reveal the influence of varying the scaled switching metric (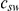), we set 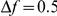 to obtain a large baseline SSA.

Figure 7B plots the mean responses to deviants and standards as a function of 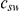 for 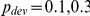. (Recall that 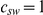 corresponds to switching as frequently as possible.) There is a clear increase in the deviant response as 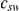 increases. The standard responses are less affected, although for 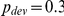, an increase in the response is visible. These trends can be interpreted as follows. Lowering 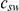 reduces the probability of transiting to the opposite state, so that sub-sequences of consecutive standards and deviants tend to be longer (*cf.* Figure 2). Decreasing 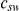 causes deviants to clump together, so that the mean deviant response drops considerably. However, standards, by virtue of appearing very often, tend naturally to form long, unbroken groups anyway, and consequently, lowering 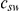 does not greatly influence the mean response to the standard. This explains why a small difference in the standard response is apparent for 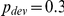, but not for 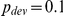. As the deviant response increases substantially with 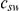, whereas the standard response increases only very little, it is clear that the SI value will increase with 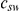, and this is indeed what is observed from the model (Figure 7C).

#### Block, sequential and random stimuli

**Figure 8:**
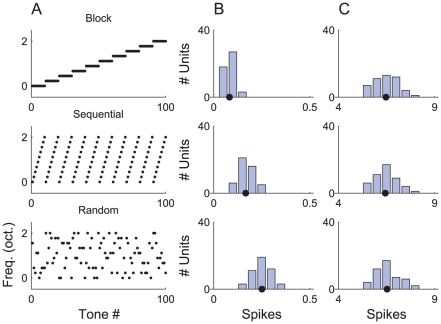
Response of the ABC model to tones presented in block, sequential and random configurations. Each row of this figure corresponds to a different presentation mode: block, sequential and random. A) Pattern of tone frequencies. B) Histograms of the mean spike count per tone for population B units. C) Histograms of the mean spike count per tone with depression on 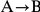 synapses disabled. The solid markers on the abscissae (in B, C) show the grand mean spike count, averaged over every tone and every unit for that condition.

We measured the mean response of units in population B to tones presented in the block, sequential and random configurations (see Methods). Histograms of the spike counts obtained are provided in Figure 8B and exhibit the same ordering as the results in Figure 4A in [2]. The mean response to tones presented in block mode was the lowest, followed by sequential mode, and then random mode. This ordering can be explained in terms of a combination of depression and the overlap in the tuning of the inputs.

In block mode, of one hundred tones, ninety are preceded by an identical tone, and nine are preceded by an adjacent frequency. Most signals are directed via depressed synapses, and the average output is small. In sequential mode, ninety tones are preceded by an adjacent frequency, and nine are preceded by a tone remote in frequency (i.e., the first tone of each ascending series); consequently, the average output is higher than in the block mode case. Finally, in random mode, it is quite improbable that a tone will be preceded by another of the same frequency (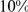) or an adjacent frequency (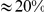). The average spike count obtained in random mode is therefore considerably higher.

Pérez-González et al. (Figure 4B in [2]) also conducted a control experiment to test whether the stimulus configuration had any effect upon the responses of units that did not exhibit SSA and found that it did not. To simulate non-habituating units, we disabled the depression in the 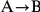 synapses (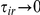). Histograms of spike counts for units in population B of the modified model are plotted in Figure 8C for each configuration and exhibit little difference. Pairwise significance tests between the three distributions confirm that the responses are unaffected by the sequence configuration once synaptic depression is removed (two-sample Kolmogorov-Smirnov test, 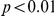). We also note that the spike counts are an order of magnitude higher when the input synapses are non-depressing, as they are in [2].

### ABD Model

#### Block, sequential and random stimuli

Block, sequential and random stimuli were submitted to the ABD model and the responses in populations B and D were recorded. The patterns in the data were similar to those obtained for the ABC model and can be understood in the same terms. A figure is included in Supplementary Text S1.

#### Markov sequences

The firing rates in populations B and D in the ABD model were measured in response to two-state Markov chain stimuli. The sequences were presented at a rate of 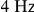, and tones had a duration of 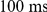. A rapid presentation rate was used, because strong SSA is required in population B in order to highlight the contribution of two layers of depressing synapses to the activity in population D. If the responses to the standards and deviants in population B are similar, then the changes in the state of the 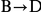 synapses are too subtle to register in population D, which tends simply to ‘inherit’ the responses of population B. A tone separation of 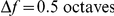 was used in these experiments.

**Figure 9:**
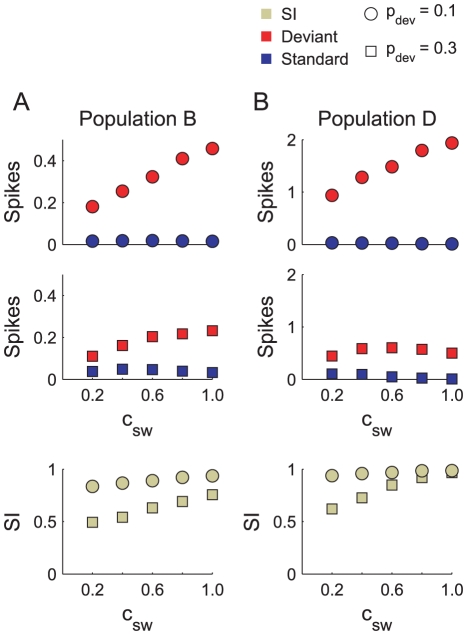
Response of the ABD model to two-state Markov chains. A) Mean spike counts per tone (top, middle panels) and SI values (bottom) measured in population B in response to Markov chains with various 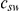 (see Methods). B) Corresponding results for population D.

Figure 9A and 9B plot the response to Markov chains in populations B and D, respectively. The first and second row plot the mean standard and deviant spike counts per tone for 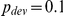 and 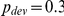, respectively. The bottom row plots the SIs derived from these spike counts. The spike counts and SIs in population B follow qualitatively identical trends to those obtained for the ABC model (Figure 7B,C). This follows from the fact that a single layer of adaptation in involved in both cases. Population D in general inherits the responses of population B, with one exceptional note. In the 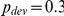 condition, for large 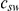 (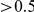), the deviant response *declines*, rather than increases. This decline can be seen as the result of depression in the 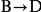 synapses. These synapses only become depressed following sufficiently high activity in population B, and this in turn can only be obtained when 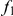 and 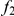 switch back and forth frequently. When 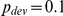, the highest probability of switching (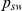) possible is 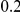, which corresponds to 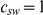 (see Methods). Evidently, this is not high enough to manifest the effects of depression in population D. However, for 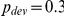 and 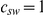, 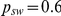: 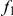 switches to 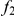 on six out of ten trials. This explains why the decline in the deviant response is present for 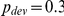 only. This analysis is also consistent with the pattern of standard responses observed.

#### Deviants amongst many standards

**Figure 10:**
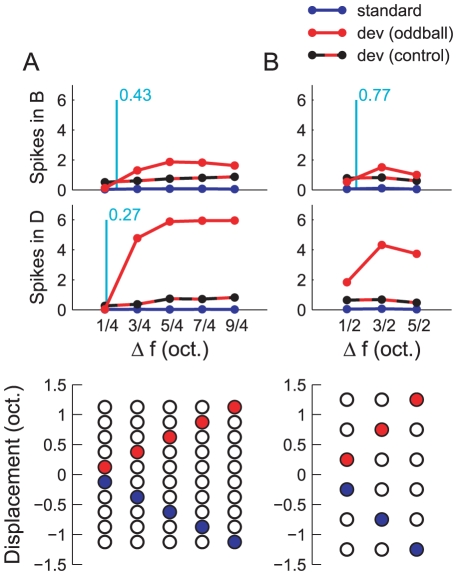
Effect on context on the response to a deviant in the ABD model. A) Mean spike counts per tone in response to deviants (red), standards (blue) and deviants embedded in many control standards (black/red striped) in population B (top panel) and D (middle panel). Each column in the bottom panel indicates the frequency of the deviant (red circle) and the standard (blue circle) used in the single standard condition. In the many standards condition, tones appear in all the positions with uniform probability, but the nominal deviant frequency is still marked by a red circle. The tone positions are spaced at intervals of 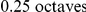. B) Results shown in the same format for six tone positions spaced at 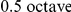 intervals. The vertical, cyan lines mark the intersections of the deviant (oddball) and deviant (control) curves, where present, according to a piecewise linear interpolation between data points.

Figure 10 shows the spike counts elicited in populations B and D in response to deviants presented in the context of both a single standard and multiple standards (see Methods). Two experiments were performed. In both experiments, sequences were presented at a rate of 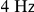, and tones had a duration of 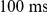.

In the first experiment, tones could appear in ten positions, spaced uniformly at 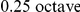 intervals over the centre of the input region. A total of 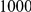 tones were presented, 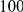 of which were deviants (i.e., 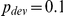). Experiments were performed in ten conditions. In the first five conditions, the deviant occupied one of the five high-frequency positions, respectively, and the remaining tones were standards, which occupied one of the five low-frequency positions. The deviants and standards were always symmetrically positioned around the centre of the input region, as illustrated in the bottom panel of Figure 10A. This resulted in standard-deviant frequency separations (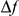) of 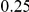, 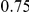, 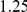, 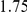 and 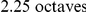. In the remaining five conditions, the deviants occupied the same respective positions as in the first five, both in terms of frequency and locations within the sequence, and the standards were distributed over the nine remaining positions with uniform probability. (The proportions were balanced so that, in each condition, a standard appeared in each non-deviant position exactly ninety times.)

The output of population B relies on a single layer of depressing synapses (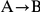). The graph in the top panel of Figure 10A shows the mean spike counts evoked in population B by standards (blue), deviants in the context of a single standard (red), and deviants in the context of many standards (black/red stripes). The responses to the standard tone are consistently the smallest. In every condition except 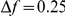, the response to a deviant was greatest when it was embedded in a single standard. This can be explained simply by cross-frequency adaptation. When the deviant and standard tones were spaced closely (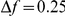), the deviant underwent adaptation due to the standard. In the many standards control condition, the deviant was exposed to less cross-frequency adaptation on average. When the deviant and standard tones were spaced further apart (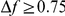), the opposite was true. The deviant underwent very little adaptation due to the single standard, but in the many standards condition, the deviant was adapted by some of the nearby control tones. This accounts for the fact that curves intersect at a location roughly equal to the input bandwidth (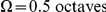; see vertical, cyan line). The shape of the curves can otherwise be explained in terms of edge effects, i.e., the fact that tones at the extreme edges do not experience adaptation from tones at frequencies on both sides.

The output of population D relies on a two layers of depressing synapses (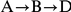). The spike counts from population D are plotted in the middle panel of Figure 10A. Many of the comments made in connection with population B in the paragraph above apply here too, as population D inherits the responses of population B to some extent. However, the excess in the response to deviants in the single standard condition, as compared to the many standards condition, is now far greater. This can be attributed to the depression of the 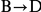 synapses when many standards are presented, and conversely, their recovery when a single standard is presented (see Methods). Despite the large effect at 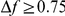, the deviants in the control condition still evoke a larger response at 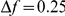, although the single standard and many standard curves now intersect at a separation lower than 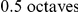.

These results demonstrate that a two-layer network is able to discriminate true deviants from tones that are simply rare, even given a frequency separation smaller than the tuning curve bandwidths of the input neurons, provided that 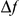 exceeds some lower limit. Nevertheless, in principle, there remains the possibility that the apparent novelty detection in population D is due to cross-frequency adaptation, as it was in population B.

In order to address this issue, a second experiment was performed, in which six tone positions were spaced at 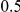-octave intervals around the centre of the input range. A total of 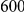 tones were presented, 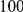 of which were deviants (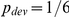). Using a broader spacing lessened the effects of cross-frequency adaptation. The adaptation channels can thereby be considered as (almost) independent. Owing to the broader spacing, only six tone frequencies were used. Experiments were thus performed in six conditions, corresponding to the three positions that the deviant could occupy crossed with the type of standard (single or many). The tone positions are illustrated in the bottom panel of Figure 10B. The results are set out, in the same format as before, in the top and middle panels of Figure 10B.

The pattern in the results is now unambiguous. The activity in population B evoked by the deviant is very similar, regardless of whether it is novel or not. In population D, the response to the true deviants is larger for all 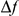 tested. Importantly, the order of the deviant responses at 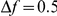 is reversed in population D. In the first experiment, to follow a rather extreme suggestion, the magnification of the true deviant response in population D could have been attributed to a monotonic non-linearity introduced by synaptic and threshold effects as it inherited from population B (e.g., perhaps spike counts greater than two in population B were greatly enhanced in population D). However, this reversal of ordering cannot be explained by these kinds of mechanisms.

## Discussion

We have proposed a model of stimulus-specific adaptation in single neurons based on the convergence of depressing synapses. The inputs to the model are Poisson processes, whose mean firing rates depend on stimulus features. In this work, the stimulus feature considered is frequency, represented on an octave scale. The firing-rate profiles are Gaussian-shaped, with bandwidths similar to auditory filters. Although we have concentrated exclusively on frequency as a stimulus feature, SSA in response to other features, such as intensity, duration and modulation, can in principle be modelled, provided that a population encoding of these features is available as input to the model.

The objective was to model the spike counts of individual neurons in response to tones embedded in various types of sequence. The model was initially calibrated to match the SSA recorded for oddball sequences in one study [9]. Stimulus configurations used in other studies were submitted to the model, including oddball sequences at other repetition rates [3], [5], [7] and sequences in which the tones were presented in blocks, in ascending sequences, and at random [2]. The trends in the results generated by the model (e.g., with respect to 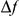, 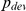 and ISI) were consistent with those measured in the respective physiological experiments, even though the latter covered a variety of brain areas, species and anaesthetic protocols.

A second contribution of the work concerns the proposed Markov stimulus paradigm. A two-state Markov chain provides a particularly useful generalisation of the oddball sequence, in that it allows the experimenter to decouple the effects of *probability* (i.e., the ratio of standards to deviants) from the effects of *switching*. In a conventional oddball sequence, the rate of switching is implicitly dictated by the deviant probability. As Markov chains have not yet been used in SSA experiments, the model also provides a direct prediction concerning the outcome of such experiments, if the explanation of SSA based on the convergence of depressing synapses is correct. Specifically, the SI for a fixed deviant probability should increase as the probability of switching increases.

The current formulation of the model allows that SSA be generated *de novo* wherever depressing synapses receive stimulus-specific inputs. A one-layer model does not account for the entire range of SSA effects observed to date, for example, instances where SSA increases as 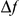 increases because the response to the standard declines [3], the tonic SSA responses observed in cortex [7], the slow adaptation component apparent over the course of an entire oddball sequence [8], the fact that SSA does not always decay with increasing SOA [4], [5] and that SSA is sometimes still strong even at very long SOAs [5]. However, a large-scale model, formed by assembling ‘modules’ of this kind, could plausibly account for the spread of SSA throughout the auditory pathway. In fact, there is evidence that neurons in IC and MGB might integrate distinct sources of SSA from multiple locations and at various latencies [3], [5]. As a first step towards this proposal, we created a two-layer model, in which one adapting process receives input from another. (This organisation is possibly reminiscent of a feed-forward process in which cortical neurons are driven by depressing thalamo-cortical synapses, which in turn are driven by adapting IC neurons, though we did not have this anatomical organisation in mind exclusively.) The neurons in the second population exhibited stronger SSA, and the “novelty component” of the response–as measured using the deviant amongst many standards control [25], [26]–was also stronger after the signal had traversed multiple depressing layers. However, some care is required when interpreting these results. An increase in SI can in part be explained by the thresholding effect of the neurons, which would be present, whether or not the intermediate synapses were depressing. It was also shown that a one-layer model can explain the fact that responses to deviants are larger when embedded in a sequence consisting of a single standard, provided the frequency separation between the deviant and standard is large enough. Arranging depressing synapses in series leads to the elicitation of novelty responses for smaller frequency separations.

Stimulus-specific adaptation in single neurons is likely to remain the subject of intense investigation in the foreseeable future, as it demonstrates a primitive form of auditory memory, upon which other novelty-related neural responses, such as auditory mismatch negativity, could build [10]. This article brings a new stimulus paradigm (Markov chains) and network architecture (two layers of adaptation, linked in series) to the attention of the research community. We suggest that adaptation should not be prematurely dismissed as the principal cause of SSA, as it is possible to capture a richer range of phenomena if adapting channels are allowed to form more complex circuits.

## Supporting Information

Supplementary Text S1 This document describes the response of the ABC model to switching oddball sequences, and block, sequential and random tone configurations. It also describes the response of the ABCD model to sequences generated by 3-state Markov chains.
(PDF)
