# Supplementary material for: A Neurocomputational Model of Stimulus-Specific Adaptation to Oddball and Markov Sequences
Source: PLoS Comput Biol. 2011 Aug 18;7(8):e1002117. doi: 10.1371/journal.pcbi.1002117 (PMC3158038; doi:10.1371/journal.pcbi.1002117)
Supplement: Supplementary Text S1 — This document describes the response of the ABC model to switching oddball sequences, and block, sequential and random tone configurations. It also describes the response of the ABCD model to sequences generated by 3-state Markov chains. (PDF) [file pcbi.1002117.s001.pdf]

A Neurocomputational Model of Stimulus-Specific Adaptation to Oddball and Markov Sequences: Supplementary Text S1

Robert Mill, Martin Coath, Thomas Wennekers, Susan L. Denham

Switching Oddball Sequences Experiment

Method

A further technique to characterise the dependence of a neuron's spike count upon the sequence stastistics is the *switching oddball paradigm* of Ulanovsky et al. [1]. In this protocol, a single sequence of tones is presented, in which the deviant tone switches back and forth between f_1 and f_2 at regular intervals. By contrast, an oddball sequence consists of two sequences of tones, and a given frequency is deviant in one sequence and standard in the other. In the switching blocks experiments performed here, one thousand blocks of twenty tones are presented, and the statistics alternate between blocks: in the odd blocks, f_1 is deviant; and in the even blocks, f_2 is deviant. In every block, $p_{dev} = 0.2$; and the ratio of standards to deviants is controlled, so there are always four deviants per block, and f_1 and f_2 appear an equal number of times over the entire sequence (i.e., ten thousand times).

The probability of a deviant and the block length used here have been carried over from the original switching blocks experiments [1]. However, we use the same tone duration (200 ms) and tone rate (1 Hz) used in the oddball experiments—a presentation rate slightly slower than that used in [1] ($\sim$ 1.36 Hz). The spike counts from the model are noisier and the adaptation effects more subtle, as in [2]; consequently, longer-term averaging is required to highlight the effect of the switching statistics. Ulanovsky et al. [1] generate two randomised blocks of twenty tones, and then repeat this "frozen sequence" twenty times. In the present experiments, each block of twenty tones is randomised independently, so that the pattern of deviants is shuffled from one block to the next, and large single trial effects are removed through averaging.

Results

This experiment measured the spikes evoked in population B of the ABC model by switching oddball stimuli. Blocks of twenty, randomly-shuffled tones were presented. Odd blocks contained four presentations of f_1 as the deviant; even blocks contained four presentations of f_2 as the deviant. The tones were separated by 0.5 octaves. Figure 1 plots the average spike counts elicited by tones f_1 and f_2 as a function of their position within an odd or even block. The model appears to adjust to the new sequence statistics within the first two or three tones of each block. Exponential curves were fitted to both the f_1 and f_2 data points in each block using a non-linear, least-squares regression, constrained so that the curves join at the block boundaries to give an unbroken, periodic rise and fall. The average time constant for the exponential trends is approximately one tone.

Ulanovsky et al. conducted the same experiment using a faster sequence and observed the same alternating pattern in the responses to the two frequencies (*cf.* [1], Figure 6C), although a close comparison reveals some differences. The rise and fall of the curves in [1], though exponential in character, have longer time courses—in terms of the change in activity per tone—than those obtained for this model. This stems, in part, from the faster tone rate used (see below); however, the majority of the difference is due to the

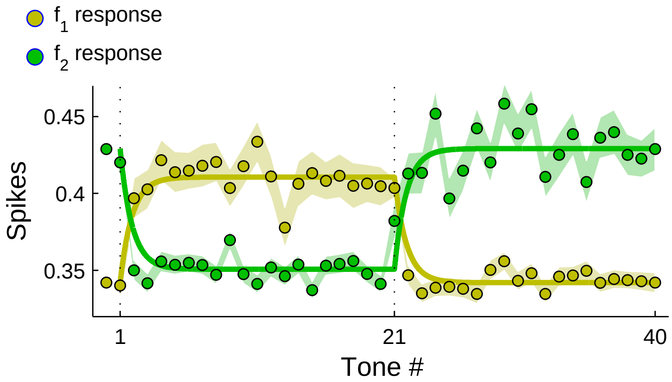

Figure 1. Spike counts averaged over switching blocks population B of the ABC model. Mean spike counts elicited by two tone frequencies, f_1 and f_2, spaced $\Delta f = 0.5$ octaves apart, during a switching blocks experiments. In "odd" twenty-tone blocks (1–20, 41–60, etc.), f_1 is deviant; in "even" twenty-tone blocks (21–40, 61–80, etc.), f_2 is deviant. The spike counts are averaged over 1000 such blocks and presented here as a single, forty-tone block. Thick curves plot periodic exponential rise and falls, fitted by a non-linear, least-squares regression. The dotted, vertical lines mark a change in the deviant frequency.

"one trial effect" [1] being much weaker in the cat auditory cortex than in the model, in which the response to a tone depends almost entirely on the tone that immediately preceded it. During a tone, the A $\rightarrow$ B synapses communicate spikes for 200 ms at a rate of up to 50 Hz. The depressing synapse model used here is depleted by the arrival of just a few spikes in rapid succession (see [3], Figure 1B). Consequently, a single tone effectively 'resets' its associated synapses completely, especially around the centre of the input rate profile. The weak effects from trials two steps earlier and beyond are presumably due to slower spiking rates at the edges of the input profiles. The shallower, asymmetric curves in [1] can be reproduced in the model by using synapses that both depress and recover more gradually. In particular, the depressing synapses should be configured in such a way that they do not depress so heavily over the course of a single tone presentation. This can be accomplished by using a smaller T_{pulse} or a larger τ_{re}, so that recovered resources are activated more briefly or at a slower rate.

Three-State Markov Chains

Method

Two-state Markov chains were introduced in the main article as a means of independently varying the probability of a deviant and the rate of switching between standards and deviants. This formulation can be readily extended to accommodate multi-tone sequences by augmenting the transition matrix to the appropriate dimension; however, this results in a large number of free parameters (quadratic in the number of tones), and these transition probabilities do not relate so naturally to qualititative and 'global' features of the sequence. One strategy to reduce parameters is to eliminate a number of transitions, i.e., set elements of M to zero; another is to specify uniform transition probabilities for certain subsets of M.

The approach taken here is to use one Markov chain to specify the switching between deviants and standards (completely parameterised by p_{dev} and c_{sw}) and a second Markov chain, effectively nested inside the "standard" state of the first, to govern switching between the many standards. The two

Table 1. Three-State Markov Chain Parameters

Cond.	Markov Process Parameters						Population B			Population D		
	p_{dev}	c_{sw}	p_{sw}	$p_{S_1\|S}$	$c_{sw\|S}$	$p_{sw\|S}$	Dv	S1	S2	Dv	S1	S2
1 (A)	0.1	0.9 *	0.18	0.5	0.5 *	0.5	0.63	0.22	0.26	2.90	0.65	0.86
2 (B)	0.1	0.9 *	0.18	0.5	0.1	0.1	0.69	0.12	0.16	3.63	0.28	0.53
3 (C)	0.1	0.9 *	0.18	0.5	0.9	0.9	0.59	0.27	0.33	2.40	0.58	0.90
4 (D)	0.1	0.9 *	0.18	$0.\dot{3}$	$0.\dot{6}$ *	$0.\dot{4}$	0.68	0.28	0.20	3.43	0.95	0.56
5 (E)	0.1	1.0	0.20	0.5	1.0	1.0	0.67	0.28	0.34	2.84	0.56	0.79
6 (F)	0.1	0.9 *	0.18	0.5	0.9	0.9	0.14	0.28	0.28	0.33	0.72	0.74

Parameters used to generate the six three-state Markov chains (see Methods). State transition diagrams for the chains are drawn in Figure 2A–F. Scaled switching metrics ($c_{sw}, c_{sw|S}$) marked with an asterisk (*) indicate natural switching probabilities for the corresponding state probabilities ($p_{dev}, p_{S_1|S}$). Note that p_{sw} and $p_{sw|S}$ are derived quantities. The final six columns list the mean spike counts in populations B and D in response to deviants, the rarer standards (S1) and the common standards (S2). The spike counts are global averages, taken over both tones and neurons.

Markov chains, though conceived of individually, actually constitute a single chain in practice. In the experiments conducted here, we use just two standard frequencies, S_1 and S_2, and their pattern of switching is controlled by two parameters: $p_{S_1|S} \leq 0.5$ sets the probability that a standard is S_1; and $0 \leq c_{sw|S} \leq 1$ sets the scaled switching metric between standards. These two quantities are analogous to p_{dev} and c_{sw} for the switching between deviants and standards. Note that S_1 is always the rarer (if any) of the two standards, and $p_{S_2|S} \equiv 1 - p_{S_1|S}$. The parametric transition matrix and stationary distribution vector are, respectively,

$$M = \begin{bmatrix} 1 - c_{sw} & c_{sw} p_{S_1|S} & c_{sw} p_{S_2|S} \\ \alpha & (1-\alpha)(1-c_{sw|S}) & (1-\alpha) c_{sw|S} \\ \alpha & (1-\alpha)\beta & (1-\alpha)(1-\beta) \end{bmatrix},$$

and

$$\pi = \begin{pmatrix} p_{dev}, & p_{std}\, p_{S_1|S}, & p_{std}\, p_{S_2|S} \end{pmatrix},$$

where $\alpha \equiv c_{sw} p_{dev}/p_{std}$, and $\beta \equiv c_{sw|S} \, (p_{S_1|S}) \, / \, (p_{S_2|S})$. (For examples of three-state transition diagrams of this kind, see Figure 2 ahead.) The three tones frequencies are spaced at octave intervals: the deviant has the highest frequency, S_2 has the lowest frequency. The tones are presented at a rate of 2 Hz.

Results

A three-state Markov chain was used to control switching between one deviant and two standards. The frequencies are spaced at octave intervals in the order $f_{S_1} < f_{S_2} < f_{dev}$. Six Markov chains, which represented qualitatively different kinds of switching, were submitted to the ABCD model. The ABCD model consists of all four populations: population B both receives inhibition from population C and excites population D via depressing synapses. Corresponding parameter sets are listed in Table 1. Figure 2A–F shows state transition diagrams for chains 1 – 6 and includes spike count histograms summarising the activity of the units in populations B and D in response to the three tone frequencies. (Data is first averaged over the population and then compiled into a histogram.)

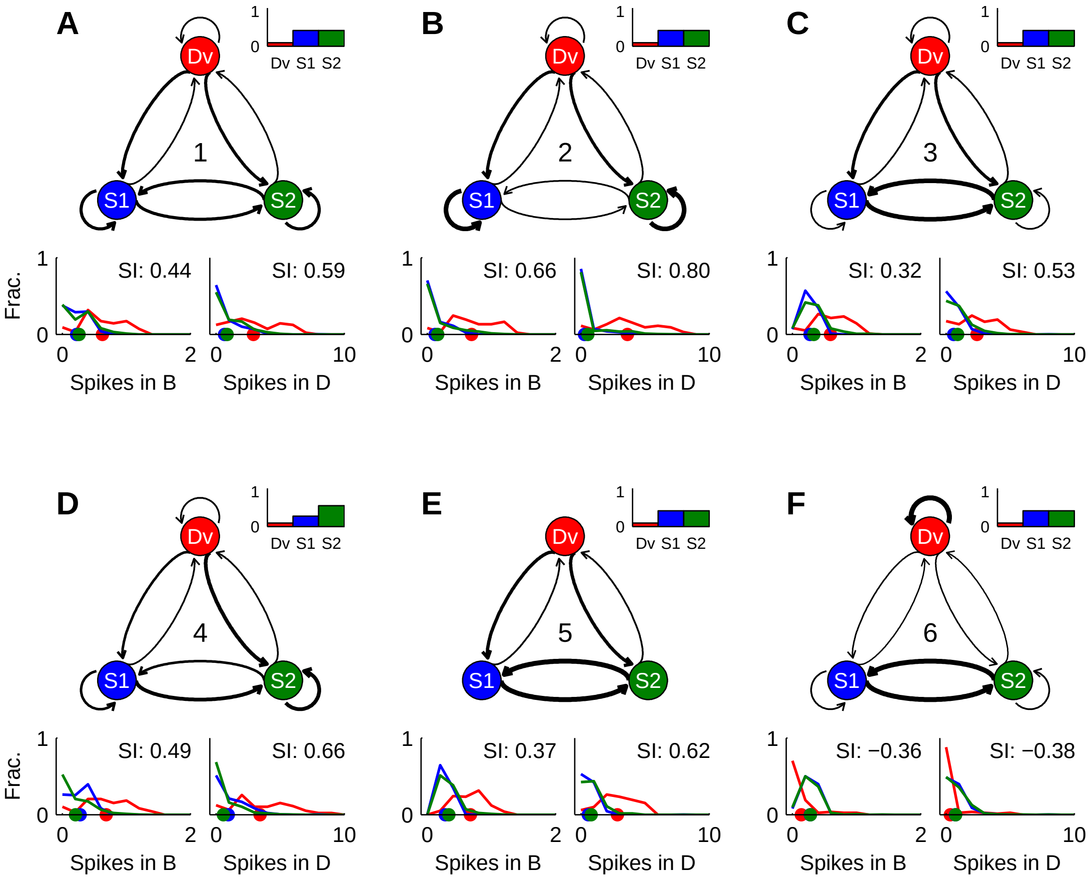

Figure 2. SSA in the ABCD model in response to three-state Markov chains. Each panel shows a state transition diagram with one deviant (Dv) and two standard (S1 and S2) states. The bar graph in the top right-hand corner plots the overall state probabilities. The bottom left-hand and right-hand corners of each panel contain histograms of spike counts for each tone type measured in populations B and D, respectively. Mean values for each histogram are plotted as solid markers on the abscissae and the corresponding figures are listed in Table 1. Mean SI values, measured for each neuron then averaged, are marked inside each plot. The qualitative characteristics of the chains are as follows (see Table 1 for parameter sets): A) equiprobable standards and natural switching between tones; B) less frequent switching between standards; C) more frequent switching between standards; D) imbalance between standards, natural switching; E) most frequent switching possible amongst deviant and standards; F) infrequent switching between deviant and standards; frequent switching within standards.

Chains 1 – 3: Varying the Rate that Standards Alternate

In chains 1 – 3, the two standards appeared with equal probability; i.e., the deviant and standards were in the ratio $dev : S_1 : S_2 = 2 : 9 : 9$. Also, $p_{dev} = 0.1$, and $c_{sw} = 0.9$ (chain 1, natural) were fixed. Only $c_{sw|S}$, the rate at which the standard switched back and forth between S_1 and S_2, was varied. Figure 2A plots the output of the model when deviant tones were embedded in a sequence of standards that switched back and forth naturally ($c_{sw|S} = 0.5$), that is, at the rate one would expect them to switch were they to be drawn independently. Figure 2B and C plot, respectively, the output of the model when the deviants were embedded in standards that switched infrequently (chain 2, $c_{sw|S} = 0.1$) and frequently (chain 3, $c_{sw|S} = 0.9$).

The mean response to deviants in population D was larger when the standards switched less often (for population-level spike count r_i when $i = c_{sw}$: $r_{0.1} > r_{0.5}$ and $r_{0.5} > r_{0.9}$; t-test with unequal variances, $p < 0.05$); but no such differences were observed in the responses of population B. When the standards switch frequently, the average activity in population B is high, and so the B $\rightarrow$ D synapses are more depressed. Consequently, deviant tones are communicated more effectively when preceded by long sequences of a single standard. The mean SI values increase in both populations when the standards switch less frequently, because the average response to a standard is reduced.

Chain 4: Two Standards with Unequal Probabilities

We now consider Markov chain 4. In this example, we modified chain 1 by changing the ratio of S_1 and S_2 within the standards. Specifically, $p_{S_1|S} = 0.3$ and $c_{sw|S} = 0.7$ (natural). The output in response to this sequence is plotted in Figure 2D and should be compared to that plotted in Figure 2A, where the two standards were equiprobable. In population B, it is clear that the spike counts are ordered ascendingly according to rarity. Furthermore, making one standard rarer than the other causes the SI to increase—by virtue of the mean standard response decreasing. However, it does not seem that this is due to changing the *ratio* of standard frequencies as such; rather, it is because a sequence in which S_1 appears less often than S_2 naturally *switches* slightly less often.

Chain 5: Highest Possible Switching Rate Amongst All Tones

Markov chain 5, drawn in Figure 2E, represents the highest possible rate of switching amongst the three tone frequencies, that is, $c_{sw} = c_{sw|S} = 1$. Note that no transition arc returns to the same state. An interesting feature of the response to this chain, when compared with the response to the chain 1 (Figure 2A), is that the SIs in populations B and D change in opposite directions. This reversal discounts the possibility that population D simply inherits its responses from population B.

In population B, the SI is lower for the chain 5 than for the chain 1. The spike count elicited by standards is higher on account of the frequent switching between standards, and the spike count elicited by deviants is also (marginally) higher, because the possibility of consecutive deviants is eliminated. Overall however, the change in the mean response to a standard is the dominant factor affecting the SI, which is now lower.

In population D, the opposite holds: the SI is greater for the chain 5 than for chain 1. The high rate of switching implies that population B is more active on average, and the B $\rightarrow$ D synapses are depressed. Population D thereby becomes less sensitive to any tone. This is particularly true for standards, whose inputs are typically strong enough to generate only a couple of spikes. Furthermore, in the super-threshold regime, the function relating the input spike rate to the output spike rate for an integrate-and-fire neuron is compressive [4]; consequently, absolute changes in the input rate have a much greater relative effect on the output at lower input rates. In summary, whilst depression reduces the response in population D to both standards and deviants, it affects standards to a greater extent, both in terms of spike rate (super-threshold) and spike probability (near-threshold), all of which culminates in an increase in the SI.

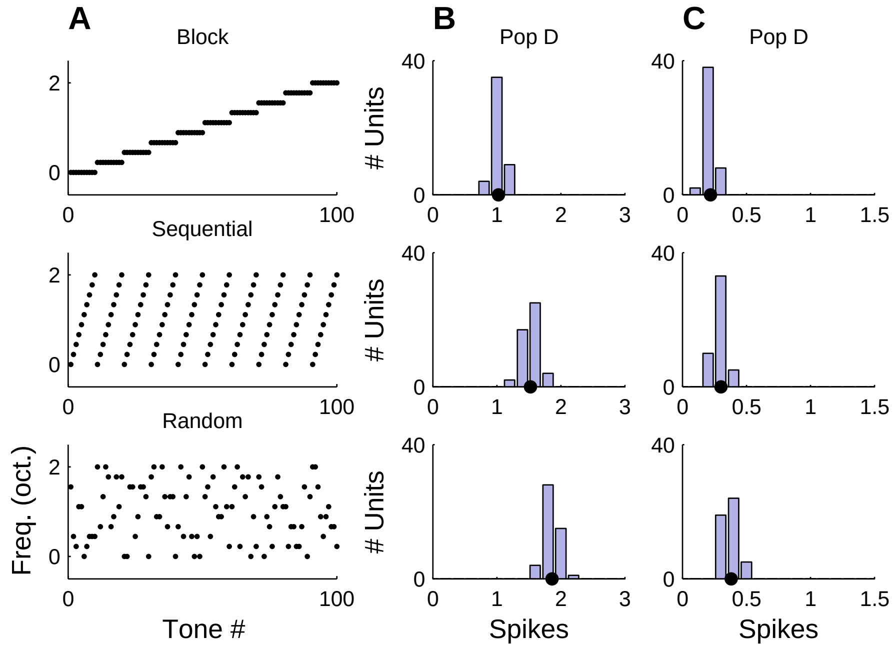

Figure 3. Response of the ABD model to tones presented in block, sequential and random configurations. Each row of this figure corresponds to a different presentation mode: block, sequential and random. A) Pattern of tone frequencies. B) Histograms of the mean spike count per tone for population B units. C) Histograms of the mean spike count per tone for the population D units. The solid markers on the abscissae (in B, C) show the grand mean spike count, averaged over every tone and every unit for that condition.

Chain 6: Clustered Deviants with Frequently Switching Standards

Finally, Markov chain 6, drawn in Figure 2F, exemplifies a situation in which deviants elicits fewer spikes on average than standards. The scaled switching metrics are set to have opposite effects: setting $c_{sw} = 0.1$ means that the sequence rarely changes back and forth between standards and deviants; but setting $c_{sw|S} = 0.9$ means that during blocks of standards, the chain switches back and forth frequently between S_1 and S_2. This alternation between standard frequencies results in a high standard response on average. During one standard, the synapses associated with the other standard are given time to recover. However, the average response to a deviant is low, because there is only a single deviant frequency—and the synapses associated with that frequency remain depressed.

Block, Sequential and Random Stimuli in the ABD model

Block, sequential and random stimuli were submitted to the ABD model and the responses in populations B and D were recorded. The patterns in the data were similar to those obtained for the ABC model and can be understood in the same terms (see section "ABC Model" in the main article.) The results are plotted in Figure 3.

References

1. Ulanovsky N, Las L, Farkas D, Nelken I (2004) Multiple time scales of adaptation in auditory cortex neurons. J Neurosci 24: 10440–10453.

2. von der Behrens W, Bäuerle P, Kössl M, Gaese BH (2009) Correlating stimulus-specific adaptation of cortical neurons and local field potentials in the awake rat. J Neurosci 29: 13837–13849.

3. Tsodyks MV, Markram H (1997) The neural code between neocortical pyramidal neurons depends on neurotransmitter release probability. Proc Natl Acad Sci USA 94: 719–723.

4. Dayan P, Abbott LF (2005) Theoretical Neuroscience: Computational and Mathematical Modeling of Neural Systems. MIT Press.
